# A Comprehensive Research on Antibiotic Resistance Genes in Microbiota of Aquatic Animals

**DOI:** 10.3389/fmicb.2018.01617

**Published:** 2018-07-26

**Authors:** Bin Hong, Yongbing Ba, Li Niu, Fei Lou, Zhaohuan Zhang, Haiquan Liu, Yingjie Pan, Yong Zhao

**Affiliations:** ^1^College of Food Science and Technology, Shanghai Ocean University, Shanghai, China; ^2^Agri-Products Quality and Safety Testing Center of Shanghai, Shanghai, China; ^3^Shanghai Engineering Research Center of Aquatic-Product Processing and Preservation, Shanghai, China; ^4^Laboratory of Quality and Safety Risk Assessment for Aquatic Products on Storage and Preservation (Shanghai), Ministry of Agriculture, Shanghai, China; ^5^Engineering Research Center of Food Thermal-Processing Technology, Shanghai Ocean University, Shanghai, China

**Keywords:** aquatic animals, antibiotic resistance genes, skin microbiota, southeast coast of China, sulfonamides, chloramphenicol

## Abstract

The occurrence of antibiotic resistance genes (ARGs) as emerging contaminants is of continued concern for human health. Antibiotics used in aquaculture have promoted the evolution and spread of ARGs. This study aimed to investigate the occurrence of 37 ARGs conferring resistance to six classes of antibiotics in 94 aquatic animals from five cities in southeast coast of China. The results showed that *flo*R, *sul*II, *sul*I, *str*B, *str*A, *aad*A, and *tet*S were identified as the prominent ARGs with the high detection frequencies ranging from 30.9 to 51.1% in total samples. Then relative expression amount of seven prominent ARGs quantified by qPCR, ranging from 0.003 to 0.065. The *tet*S was the most abundant ARG among the seven ARGs. Though *aad*A was the second highest detection frequency of ARGs, it was the lowest expression amount ARG. The occurrences and abundances of ARGs in freshwater aquatic animals were greater than those in marine, reflecting the discrepancy of cultivation pattern between the freshwater and marine aquaculture. Shanghai was considered as the most prevalent site with 16 ARGs, and Ningbo merely contained 9 ARGs without of β-lactam ARGs and quinolone ARGs, showing variations of ARGs with geographical location. Eight kinds of sulfonamides and one chloramphenicol residues were further measured in samples from Shanghai. Interestingly, no target antibiotics were found, but sulfonamides resistance genes (*sul*I, *sul*II) and chloramphenicol resistance genes (*flo*R) persisted at aquatic animals in the absence of selection pressure. Our research firstly shows comprehensive information on the ARGs in skin microbiota of aquatic animals, which could provide useful information and a new insight for better understanding on the ARGs dissemination in aquatic animals.

## Introduction

Antibiotic resistance genes (ARGs), emerging environmental contaminants, draw an increasingly attention due to their huge risk to human health (Pruden et al., 2006). ARGs encoding resistance to a broad range of antibiotics have been found to be able to spread among bacteria via horizontal gene transfer (HGT), thus aggravating ARGs dissemination (Thomas and Nielsen, 2005; Bellanger et al., 2014). In addition, antibiotic-resistance bacteria that are associated with wild animals is correlated with the proximity of the animals (and the bacteria) to human populations. Migratory animals are important contributors to the widespread dissemination of ARGs (Allen et al., 2010). Many bacteria, especially human commensal bacteria and pathogens, have been reported to be multi-drug resistant (Hatha et al., 2005) and capable of transferring their resistance determinants among environmental bacteria of different genera (Agersø and Petersen, 2007; Guglielmetti et al., 2010), which results in a huge adverse effect to human health. Also, the World Health Organization (WHO) pointed out that antibiotic resistance already had become one of the greatest threats to aquatic food and, consequently, global public health through the food chain^1^.

In 2016, the total number of aquatic products was nearly 70 million tons in China, an increase of 3.69% than last year, of which aquaculture products accounted for 73% (CAP, 2016). To sustain the rapid and steady growth of this industry, it’s necessary to obtain the profit of aquaculture by using antibiotics to ensure high animal growth and low infectious diseases. The broad-spectrum antibiotics had been vastly used in aquaculture and husbandry since they were found that they could be used not only for the prevention and treatment of infectious diseases, but also for promotion of animal growth and feed efficiency (Gustafson and Bowen, 1997). In China, the annual usage of raw antibiotic ingredients for both human and agriculture is up to 180,000 t (Zheng et al., 2012). Approximately 70% of these antibiotics are excreted in the unaltered forms, which are eventually discharged into the water environment in a variety of ways, such as through the disposal of sewage, hospital wastewater and animal waste (Kümmerer and Henninger, 2003; Yang et al., 2011). All these scenarios and the artificially added antibiotics posed a great threat to both freshwater and marine aquaculture.

Southeast coast of China, typical subtropical climate, is one of the fastest developing and most highly urbanized regions in China. However, to our knowledge, most studies to date have focused on antibiotic contamination in one area alone, like Shanghai, Hangzhou, Ningbo, Fuzhou, and Xiamen (Jiang et al., 2011; Li et al., 2011; Ji-Bing and Hu, 2012; Shen et al., 2014; Tang et al., 2017), very few comprehensive researches on ARGs in microbiota of aquatic animals have been paid attention to in these area together (Xu et al., 2007). In addition, many reports were available on the occurrence and abundance of ARGs and antibiotic residues in water and sediment in aquaculture environment (Gao et al., 2012; Cheng et al., 2013; Chen et al., 2017; Nakayama et al., 2017). However, there are very few reports on ARGs and antibiotic residues in aquatic animals, therefore, it is necessary to investigate ARGs and antibiotic residues in aquatic animals of this area.

Compared to many previous researches on aquatic animal gut microbiota (Xiong et al., 2015; Muziasari et al., 2016b; Fu et al., 2017), to the best of our knowledge, this is a first comprehensive study on antibiotics in aquatic animal skin microbiota. The purpose of this study aimed to investigate the occurrence of 37 ARGs conferring resistance to six classes of antibiotics in 94 aquatic animals from five cities (Shanghai, Hangzhou, Ningbo, Fuzhou, Xiamen) located in southeast coastal area of China. Eight kinds of sulfonamides and one chloramphenicol residues were further measured in samples from Shanghai. This investigation would give us a new insight on the ARGs dissemination in aquatic animals, which might be paid attention to.

## Materials and Methods

### Sample Sites and Sample Collection

Sixteen species of 94 aquatic animals were collected in August to October 2015 from five southeast coastal cities of China including Shanghai, Hangzhou, Ningbo, Fuzhou, Xiamen. The samples were obtained from local markets. After sampling, they were stored at -80°C for further analysis. A summary of the sampling and reference sites is shown in **Table 1**.

**Table 1:** The sites and species of 94 samples collected in this study.

Species		The number of samples					
		
		Shanghai	Hangzhou	Ningbo	Fuzhou	Xiamen	Total
Freshwater aquatic animals (62)	Largemouth bass (*Micropterus salmoides*)	3	3	2	1	3	12
	Bighead carp (*Hypophthalmichthys nobilis*)	1	1	3	2	1	8
	Common carp (*Cyprinus carpio*)	2	–	–	–	–	2
	Mandarin fish (*Siniperca chuatsi*)	3	2	1	1	1	8
	Crucian carp (*Carassius carassius*)	8	2	1	3	1	15
	Snakehead (*Channa argus*)	3	1	1	–	–	5
	Yellowhead catfish (*Pelteobagrus fulvidraco*)	2	1	–	1	–	4
	Grass carp (*Ctenopharyngodon idella*)	2	2	2	2	–	8
Marine aquatic animals (32)	Turbot (*Scophthalmus maximus*)	4	1	1	1	1	8
	Pompano (*Trachinotus ovatus*)	–	–	–	–	3	3
	Large yellow croaker (*Larimichthys crocea*)	3	1	–	–	–	4
	Yellow grouper (*Epinephelus awoara*)	–	–	2	2	3	7
	Japanese sea bream (*Pagrus major*)	–	–	2	–	1	3
	Red drum (*Sciaenops ocellatus*)	–	–	–	1	1	2
	Pacific white shrimp (*Litopenaeus vannamei*)	1	1	–	1	–	3
	Giant tiger prawn (*Penaeus monodon*)	1	–	–	1	–	2
Total		33	15	15	16	15	94


### DNA Extraction

All aquatic animals were dissected under aseptic conditions. The DNA of skin bacteria was extracted by using TIANamp Bacteria DNA Kit (Tiangen Biotech Beijing Co., Ltd., China) according to the manufacturer’s instructions. There was a slight modification in extraction method, where the incubation time in lysozyme was increased to 1 h and the incubation time in proteinase K was increased to 2 h (Ye et al., 2013). The quality of extracted DNA was verified by 2% agarose gel electrophoresis, and the concentrations of DNA were measured by multifunction enzyme labeling instrument (BioTek Synergy2, American). At last, the DNA was stored at -20°C prior to PCR analysis.

### Detection of ARGs

The presence of 37 ARGs were identified by PCR, including β-lactam ARGs (CARB, SHV, SHV-5, *amp*C, *mec*A), tetracycline ARGs (*tet*A, *tet*B, *tet*M, *tet*O, *tet*Q, *tet*S, *tet*W, *tet*K), aminoglycoside ARGs (*aph(2′)-Ib*, *str*A, *str*B, *aad*A, *aad*E, *aac(6′)-Ib*, *arm*A, *rmt*B), quinolone ARGs (*qnr*S, *aac(6′)-Ib-cr*, *qnr*A, *qnr*C, *qnr*D, *par*C, *qnr*B), chloramphenicol ARGs (*cat*I, *cat*II, *cat*III, *cat*IV, *flo*R), and sulfonamide ARGs (*sul*I, *sul*II, *sul*III, sulA). Primers of all target ARGs (Supplementary Table S1) were synthesized based on our previous study (Lou et al., 2015; Li et al., 2017). PCR results were sequenced by Sangon Biotech (Sangon Biotech, Shanghai, China) and analyzed by the National Center for Biotechnology Information website (NCBI)^2^.

### Quantification of 16S rRNA and ARGs

Identified prominent ARGs (*tet*S, *str*A, *str*B, *aad*A, *sul*I, and *sul*II) were further quantified by using SYBR Green quantitative real-time PCR (qPCR). The qPCR assays were performed on ABI7500 (Applied Biosystems, United States), and the reaction system (20 μL) included 10 μL SYBR Green Premix (Sangon Biotech, Shanghai, China), 0.3 μL of each primer and 2 μL of template DNA. The detail qPCR program was as follows: 1 min at 95°C, followed by 40 cycles of 15 s at 95°C, 30 s at annealing temperature (Supplementary Table S2), 30 s at 72°C. Resistance genes (*tet*S, *str*A, *str*B, *aad*A, *sul*I, and *sul*II) and reference gene (16S rDNA) were cloned into TA vector and then transfected into *Escherichia coli* DH5α (Tiangen Biotech Beijing Co., Ltd., China). Plasmids carrying target genes were used to generate calibration curves, and negative controls were performed for each run. The qPCR efficiencies ranged from 90 to 110% with *R*^2^-values greater than 0.99 for all calibration curves, and the analytical conditions were described in previous study (Chen et al., 2017).

### Antibiotic Residues Analysis

The antibiotic standards, including eight kinds of sulfonamides (sulfadiazine, sulfathiazole, sulfamethyldiazine, sulfamethazine, sulfamethoxazole, sulfadoxine, sulfisoxazole, and sulfaquinoxaline) and chloramphenicol, were obtained from ALADDIN Chemical Co., Ltd. (Shanghai, China). The antibiotic solutions were prepared at a concentration of 500 μg/ml in methyl alcohol and stored at -80°C for further experiment.

Thirty-three aquatic samples from Shanghai were selected to analyze antibiotic residues. All aquatic animals were dissected under aseptic conditions and muscles were used to detect antibiotic residues. Ten grams of aquatic animals were selected, and the experimental samples were placed in 50 ml centrifuge tubes and extracted by Mellvmince-EDTA buffer. The concentration of target antibiotics in aquatic animals was measured by high performance liquid chromatography-tandem mass spectrometry (HPLC-MS/MS) system (Manchester, United Kingdom). The analytical conditions were described in previous study (Hon et al., 2016).

### Data Analysis

The occurrences and abundances of ARGs were performed with OriginPro 9.1 (OriginLab, United States). The average values were calculated by Excel 2016 (Microsoft, United States), Heatmap analysis was conducted by HemI version 1.0 (Heatmap Illustrator, China).

## Results

### ARGs Diversity in Aquatic Animals

**FIGURE 1:**
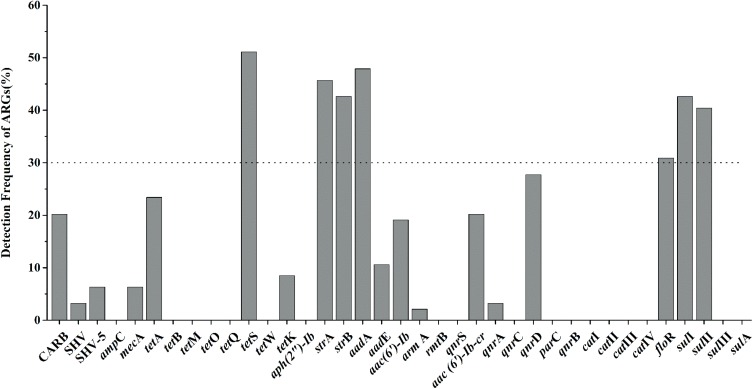
The frequencies of 37 antibiotic resistance genes (ARGs) conferring resistance to six classes of antibiotics in total aquatic animals. (The dotted line shows seven major genes with a detection rate of more than 30%).

Nineteen of thirty-seven target ARGs were found with detection frequencies ranging from 2.1 to 51.1% in total aquatic animals (CARB, SHV, SHV-5, *mec*A, *tet*A, *tet*S, *tet*K, *str*A, *str*B, *aad*A, *aad*E, *aac(6′)-Ib*, *arm*A, *aac(6′)-Ib-cr*, *qnr*A, *qnr*D, *flo*R, *sul*I, and *sul*II) (**Figure 1**). The *flo*R, *sul*II, *sul*I, *str*B, *str*A, *aad*A, and *tet*S were identified as the prominent ARGs with the high detection frequencies ranging from 30.9 to 51.1%, suggesting a serious condition conferring resistance to tetracyclines, aminoglycosides, chloramphenicol, and sulfonamides in aquatic animals. All categories were detected in the six categories ARGs. For β-lactam ARGs, four encoding β-lactamase genes, CARB, SHV, SHV-5 and *mec*A, were detected with low detection frequencies ranging from 3.2 to 20.2%. Three (*tet*A, *tet*S, *tet*K) of eight tetracycline ARGs were found with the detection frequency ranging from 8.5 to 51.1%. It is noteworthy that *tet*S exhibited the highest frequently detection of the 37 ARGs in our study (51.1%, *n* = 94). Six of eight aminoglycoside ARGs were found ranging from 2.1 to 47.9%. The q*nr*D was the predominant ARG detected in seven quinolone ARGs, with the detection frequency of 27.7%, followed by *aac(6′)-Ib-cr* and *qnr*A (20.2 and 3.2%). The *flo*R was the only chloramphenicol ARGs with the high detection frequency (30.9%, *n* = 94). In sulfonamides ARGs, *sul*I, and *sul*II exhibited the high detection frequencies of 41.5 and 40.4%, but *sul*III and *sul*A were not observed in any samples.

### The Seven Prominent ARGs Relative Expression Amount Analysis

**FIGURE 2:**
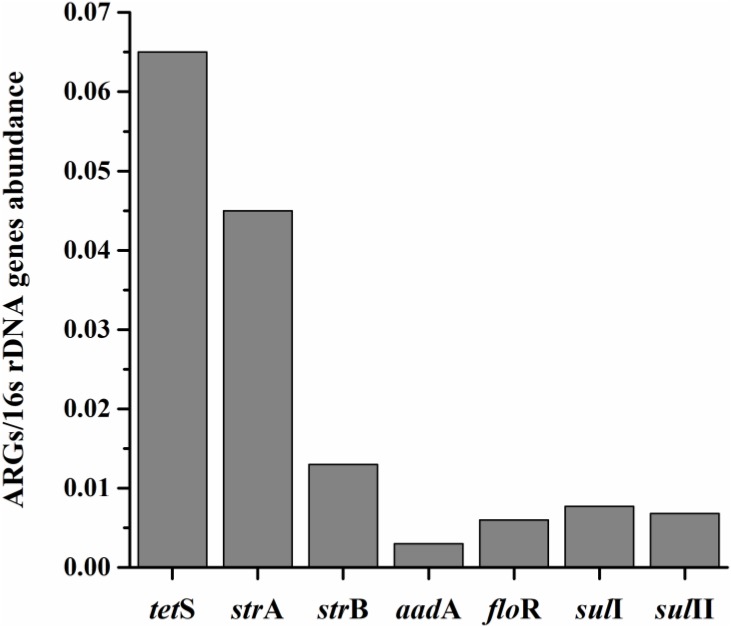
The average relative expression (to internal reference gene 16S rDNA) of seven prominent ARGs (*tet*S, *str*A, *str*B, *aad*A, *sul*I, and *sul*II) in total aquatic samples.

The average relative expression (to internal reference gene 16S rDNA) of seven prominent ARGs in total aquatic animals decreased as: *tet*S > *str*A > *str*B > *sul*I > *sul*II > *flo*R > *aad*A, ranging from 0.003 to 0.065 (**Figure 2**). The *tet*S was the highest abundant ARG in aquatic animals among the seven prominent ARGs, with the average relative expression in *tet*S positive samples 1.27 × 10^-1^/16S rDNA (the relative abundance ranged from 2.00 × 10^-4^ to 9.46 × 10^-1^/16S rDNA, *n* = 48). Slight gaps were observed in the average relative expression of *str*B, *flo*R, *sul*I, and *sul*II. Though *aad*A was the second highest detection frequency of 37 ARGs, it was the lowest abundant ARG. The average relative expression in *aad*A positive samples was 6.30 × 10^-3^/16S rDNA (*n* = 45). Overall, *tet*S was also the highest frequently detection of 37 ARGs.

### Comparison ARGs Diversity Between Freshwater and Marine Aquatic Animals

**FIGURE 3:**
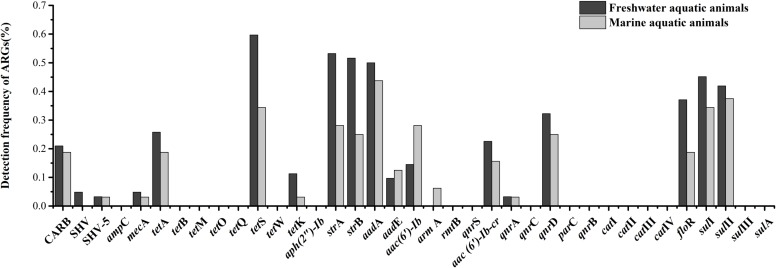
The frequencies of 37 ARGs detected in freshwater aquatic animals and marine aquatic animals.

Eighteen of thirty-seven target ARGs were found in freshwater aquatic animals with detection frequencies ranging from 3.2 to 59.7%, also, 18 genes were found in marine aquatic animals with detection frequencies ranging from 3.1 to 43.8% (**Figure 3**). The *tet*S was the highest detection of the 37 ARGs in freshwater aquatic animals (59.7%, *n* = 62), but the *aad*A was the highest detection of the 37 ARGs in marine aquatic animals (43.8%, *n* = 32). Interestingly, the SHV (4.8%, *n* = 62) of β-lactam ARGs was the only one existed in freshwater aquatic animals, and the *arm*A (6.3%, *n* = 32) of aminoglycoside ARGs was the only one existed in marine aquatic animals.

**FIGURE 4:**
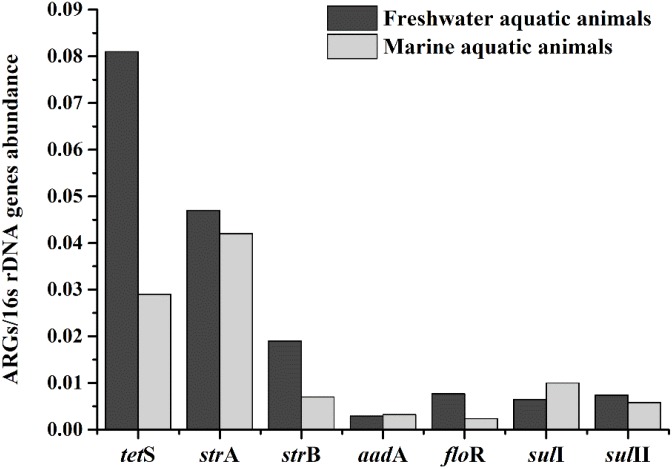
The average relative expression of seven prominent ARGs (*tet*S, *str*A, *str*B, *aad*A, *sul*I, and *sul*II) in freshwater aquatic animals and marine aquatic animals.

The relative expression amount of seven prominent ARGs in freshwater aquatic animals also were higher than those in marine aquatic animals (**Figure 4**). The average relative abundance of freshwater aquatic animals was 0.025, being approximately 1.73 times greater than those in marine aquatic animals. The *tet*S was the highest abundant ARG in freshwater aquatic animals, but the *str*A was the highest abundant ARG in marine aquatic animals. Overall, the occurrences and abundances of ARGs in freshwater aquatic animals were greater than those in marine aquatic animals.

### ARGs Diversity With Geographical Location

**FIGURE 5:**
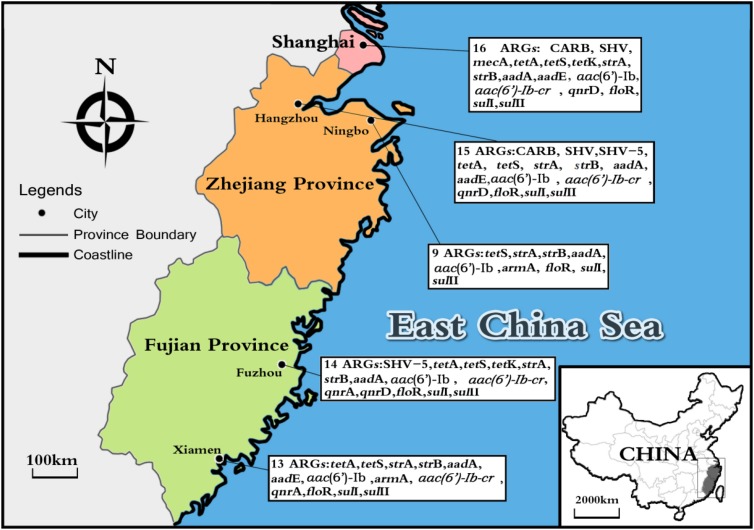
The sites of total aquatic animals collected in five southeast coastal cities of China (Shanghai, Hangzhou, Ningbo, Fuzhou, and Xiamen) and ARGs diversity distribution.

**FIGURE 6:**
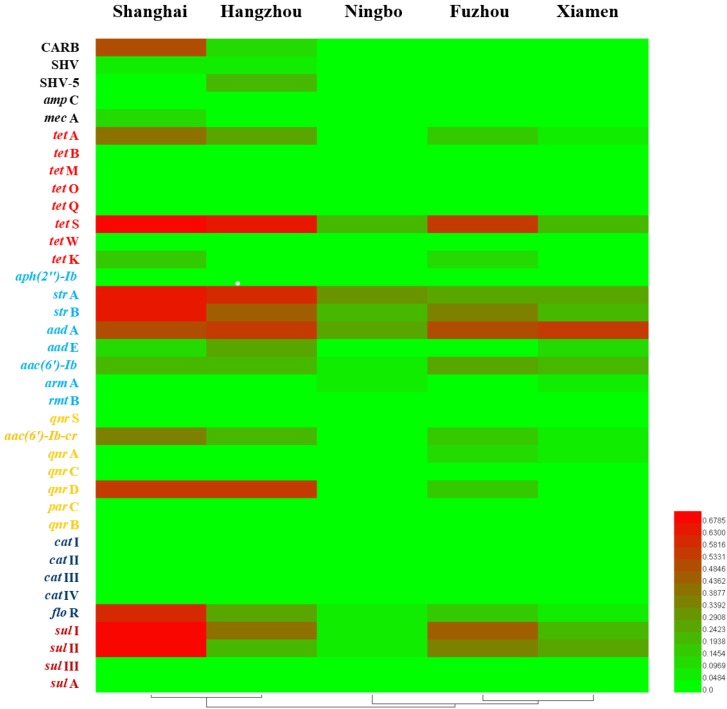
The detection frequencies of 37 ARGs in five southeast coastal cities of China (Shanghai, Hangzhou, Ningbo, Fuzhou, and Xiamen).

Considering ARGs presence in different cities (**Figure 5**), Shanghai was considered the most prevalent site with sixteen ARGs (CARB, SHV, *mec*A, *tet*A, *tet*S, *tet*K, *str*A, *str*B, *aad*A, *aad*E, *aac(6′)-Ib*, *aac(6′)-Ib-cr*, *qnr*D, *flo*R, *sul*I, and *sul*II), whereas Ningbo contained nine ARGs (*tet*S, *str*A, *str*B, *aad*A, *aac(6′)-Ib*, *arm*A, *flo*R, *sul*I, and *sul*II), with only nine ARGs and none of β-lactam ARGs and quinolone ARGs, showing variations of ARGs with geographical location (**Figure 6**). All the prominent seven resistance genes *tet*S, *str*A, *str*B, *aad*A, *flo*R, *sul*I, and *sul*II were observed in five cities samples.

**FIGURE 7:**
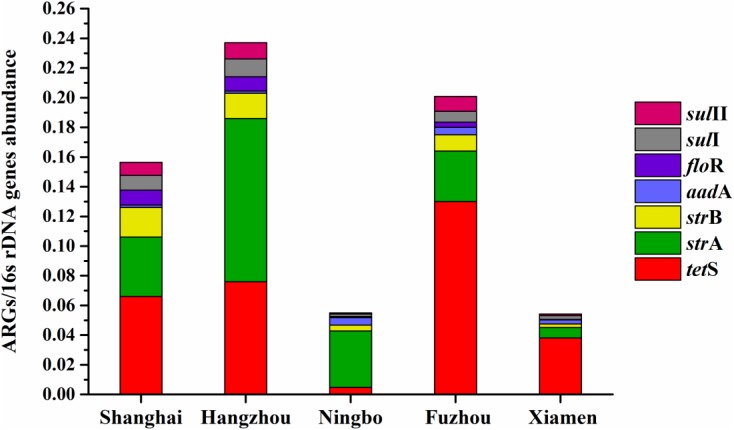
The average relative expression of seven prominent ARGs (*tet*S, *str*A, *str*B, *aad*A, *sul*I, and *sul*II) in five southeast coastal cities of China.

The average relative expression of *tet*S was the most abundant ARG in Shanghai (0.066), Fuzhou (0.13), and Xiamen (0.038), but the *str*A was the most abundant ARG in Hangzhou (0.11) and Ningbo (0.11). The total concentration of seven ARGs in Hangzhou is the highest abundant in five cities followed by Fuzhou, Shanghai, Ningbo, and Xiamen (**Figure 7**). This result reflected a fact that aquatic animals in the southeast coastal area of China contained various ARGs, furthermore, *tet*S, *str*A, *str*B, *aad*A, *flo*R, *sul*I, and *sul*II posed a leading position in ARG contaminations.

### Relationship Between Antibiotic Residues and ARGs

Shanghai was considered as the most prevalent site with 16 ARGs in five cities, so thirty-three samples from Shanghai were chosen to analyze antibiotic residues, including sulfadiazine, sulfathiazole, sulfamethyldiazine, sulfamethazine, sulfamethoxazole, sulfadoxine, sulfisoxazole, sulfaquinoxaline, and chloramphenicol. Though chloramphenicol ARGs (*flo*R) and sulfonamide ARGs (*sul*I and *sul*II) were dominant ARGs in aquatic animals in our study, it was worth noting that no target antibiotics were detected (the detection limit of target antibiotics were 1.0 μg/kg). Result showed that *flo*R, *sul*I, and *sul*II persist at aquatic animals in the absence of selection pressure (**Table 2**).

**Table 2:** Relationship between antibiotic residues and ARGs in Shanghai samples.

Species		Numbers	Sulfonamides	Chloramphenicol	*sul*I	*sul*II	*flo*R
Freshwater aquatic animals	Largemouth bass (*Micropterus salmoides*)	3	-	-	+	+	+
	Bighead carp (*Hypophthalmichthys nobilis*)	1	-	-	+	+	+
	Common carp (*Cyprinus carpio*)	2	-	-	-	-	+
	Mandarin fish (*Siniperca chuatsi*)	3	-	-	+	+	+
	Crucian carp (*Carassius carassius*)	8	-	-	+	+	+
	Snakehead (*Channa argus*)	3	-	-	+	+	+
	Yellowhead catfish (*Pelteobagrus fulvidraco*)	2	-	-	+	+	-
	Grass carp (*Ctenopharyngodon idella*)	2	-	-	-	-	+
Marine aquatic animals	Turbot (*Scophthalmus maximus*)	4	-	-	+	+	+
	Large yellow croaker (*Larimichthys crocea*)	3	-	-	+	+	+
	Pacific white shrimp (*Litopenaeus vannamei*)	1	-	-	+	+	+
	Giant tiger prawn (*Penaeus monodon*)	1	-	-	-	+	-


*+*Detected*. -*Not detected*.*

## Discussion

Antibiotic resistance genes can pose a threat to food security and human health via various pathways (Cabello, 2004; Liu et al., 2014; Aydin et al., 2015; Amandine et al., 2016). Most ARGs acquired through HGT had been originated in environmental microbiota (Martínez, 2008; Wang et al., 2012), and ARGs disseminated from farming source to reared organisms (Su et al., 2017). In addition, many previous studies mainly focused on sediments, water and aquatic animal gut microbiota (Khan et al., 2013; Fu et al., 2017; Gao et al., 2018), this study firstly reflected the occurrence of 37 ARGs conferring resistance to six classes of antibiotics in skin microbiota of aquatic animals from southeast coastal area of China, which provides a comprehensive profile on ARGs in microbiota of aquatic animals.

In our study, occurrence of 37 ARGs were investigated in 94 aquatic animals from five cities in southeast coast of China. The *tet*A, *tet*K, and *tet*S were found with the detection frequencies ranging from 8.5 to 51.1%, since wide usage of tetracyclines in environment might address this case (Cheesanford et al., 2001; Heuer et al., 2009; Yan et al., 2018a,b). In addition, the *str*A, *str*B, *sul*I, and *sul*II were prevalent in this study, which was consistent with the previous findings about the occurrence of ARGs obtained from aquaculture sediment and aquaculture water (Gao et al., 2012; Xiong et al., 2015; Muziasari et al., 2016a), and also similar to previous reports in swine farms, broiler feedlots and domestic sewage (Zhu et al., 2013; Chen et al., 2016; He et al., 2017). Results suggest that antibiotic usage pose a serious threat to the aquaculture and water environment, consequently, to public health through the food chain. The *tet*B, *tet*M, *tet*O, *tet*Q, and *tet*W were negative, this result is different from those observed in aquaculture farms, where the *tet*M, *tet*W, *tet*Q, *tet*O were found in aquaculture farm sediments and water (Ma et al., 2014; Su et al., 2014). The occurrence of ARGs obtained from aquatic animal skin microbiota is inconsistent with the sediments, water and aquatic animal gut (Khan et al., 2013; Fu et al., 2017; Gao et al., 2018). In this study, aquatic animal skin microbiota may be another important niche for dissemination of ARGs. And the mobility of aquatic animal may facilitate the proliferation and propagation of ARGs in water environment.

A previous study have investigated the heavy metal in marine aquatic animal and freshwater aquatic animal in south China (Cheung et al., 2008), however, no study focused on ARGs differences between freshwater aquatic animals and marine aquatic animals. Results showed that occurrences and abundances of ARGs in freshwater animals were greater than those in marine aquatic animals, reflecting the importance of freshwater aquaculture environment in ARGs emergence. The discrepancy of cultivation pattern might address this phenomenon. Compared to marine aquatic animals, aquatic animals in freshwater aquaculture environment could easily receive more antibiotic residues from hospital effluents, plant sewage, urban wastewater and so on. Thus, freshwater animals harbored more ARGs.

We also analyzed the occurrence and abundance of ARGs in five cities. Distinctly, the detection frequencies of ARGs among various geographical locations were different. Shanghai was considered as the most prevalent site with 16 ARGs. Since Shanghai is one of the most urbanized and developed cities in China, and the usage of antibiotics in Shanghai is more than the other four cities (Jiang et al., 2011; Liu et al., 2017). When come to the geographical location differences, anthropogenic activity could address the situation, which might reflect diversity in antibiotic usage in five cities.

The application of antibiotics in aquaculture was one of the important reasons for improving the antibiotics resistance and enhancing the concentration of ARGs in the aquaculture environment (Cesare et al., 2013; Liu et al., 2017). Wide usage of chloramphenicol and sulfonamides in aquaculture has been reported (Li et al., 2006; Heuer et al., 2009; Liu et al., 2017). So, our study simultaneously investigated the concentrations of chloramphenicol and sulfonamides in aquatic animals by HPLC-MS/MS. The prominent sulfonamide ARGs, *sul*I, and *sul*II were present in aquatic animals but no sulfonamides were detected, this result suggested that sulfonamide-resistance genes *sul*I and *sul*II persisted at aquatic animals in the absence of selection pressure. Tetracycline resistance genes and aminoglycoside resistance genes were present at fish farms without presence of the respective antibiotics (Tamminen et al., 2011; Muziasari et al., 2014). The *flo*R was the only one chloramphenicol resistance gene detected, but chloramphenicol was not detected in this study, because the Ministry of Agriculture in People’s Republic of China banned the use of chloramphenicol in food producing animals in 2002 (No. 193 Bulletin, 2002). Our results suggest that chloramphenicol resistance gene are highly persistent and do not disappear from aquaculture environment, even after several years without chloramphenicol use.

## Conclusion

Overall, our study is a first comprehensive research on the occurrences and abundances of 37 ARGs conferring resistance to six classes of antibiotics in skin microbiota of aquatic animals from five cities (Shanghai, Hangzhou, Ningbo, Fuzhou, and Xiamen) located in southeast coastal area of China. Shanghai was considered as the most prevalent site with 16 ARGs, and the geographical location differences could be contributed to anthropogenic activity. The occurrences and abundances of ARGs in freshwater animals were greater than those in marine aquatic animals, which reflect the discrepancy of cultivation pattern between freshwater and marine aquaculture pattern. Interestingly, no target antibiotics were found, sulfonamides resistance genes (*sul*I, *sul*II) and chloramphenicol resistance genes (*flo*R) persisted at aquatic animals in the absence of selection pressure. Our study suggests that aquatic animal skin microbiota contribute to the spread of ARGs in water environments, which could provide useful information for better understanding of the contamination caused by ARGs and antibiotics. Results show that ARGs pose an alarmingly serious risk in aquatic animals, which should be paid more attention to by local government.

## Author Contributions

BH, YB, and LN contributed equally to writing the draft manuscript and carrying out the experiment. FL assisted in completing the experiments. YZ, YP, HL, and ZZ provided support for experimental design and edited the final manuscript.

## Conflict of Interest Statement

The authors declare that the research was conducted in the absence of any commercial or financial relationships that could be construed as a potential conflict of interest.

## Footnotes

1 http://www.who.int/mediacentre/factsheets/antibiotic-resistance/en/

2 http://www.ncbi.nlm.nih.gov/BLAST
