# Supplementary material for: A Comprehensive Research on Antibiotic Resistance Genes in Microbiota of Aquatic Animals
Source: Front Microbiol. 2018 Jul 26;9:1617. doi: 10.3389/fmicb.2018.01617 (PMC6070771; doi:10.3389/fmicb.2018.01617)
Supplement: Supplementary file 1 [file Table_1.doc]

**Table S1** Thirty-eight antibiotic resistance genes (ARGs) used in this study and their PCR primes

| Gene | Primer | Sequence of primers (5–3’) | Amplification length (bp) | Annealing temperature (°C) | Reference |
| --- | --- | --- | --- | --- | --- |
| β-lactam ARGs | | | | | |
| CARB | FW | CAAGTACTTTYAAAACAATAGC | 534 | 46 | Jiang et al., 2013 |
|  | RV | GCTGTAATACTCCKAGCAC |
| SHV | FW | GCGAAAGCCAGCTGTCGGGC | 304 | 62 | Jiang et al., 2013 |
|  | RV | GATTGGCGGCGCTGTTATCGC |
| SHV-5 | FW | TGTTAGCCACCCTGCCGCT | 825 | 61 | Jiang et al., 2013 |
|  | RV | GTTGCCAGTGCTCGATCAG |
| *amp*C | FW | GTGACCAGATACTGGCCACA | 822 | 61 | Jiang et al., 2013 |
|  | RV | TTACTGTAGCGCCTCGAGGA |
| *mec*A | FW | TAATAGTTGTAGTTGTCGGGTTTG | 733 | 61 | Jiang et al., 2013 |
|  | RV | TAACCTAATAGATGTGAAGTCGCT |
| Tetracycline ARGs | | | | | |
| *tet*(A) | FW | GCGCTNTATGCGTTGATGCA | 387 | 62 | Jiang et al., 2013 |
|  | RV | ACAGCCCGTCAGGAAATT |
| *tet*(B) | FW | TACGTGAATTTATTGCTTCGG | 206 | 60 | Jiang et al., 2013 |
|  | RV | ATACAGCATCCAAAGCGCAC |
| *tet*(M) | FW | ACAGAAAGCTTATTATATAAC | 171 | 60 | Jiang et al., 2013 |
|  | RV | TGGCGTGTCTATGATGTTCAC |
| *tet*(O) | FW | ACGGARAGTTTATTGTATACC | 171 | 60 | Jiang et al., 2013 |
|  | RV | TGGCGTATCTATAATGTTGAC |
| *tet*(Q) | FW | AGAATCTGCTGTTTGCCAGTG | 169 | 63 | Jiang et al., 2013 |
|  | RV | CGGAGTGTCAATGATATTGCA |
| *tet*(S) | FW | GAAAGCTTACTATACAGTAGC | 169 | 50 | Jiang et al., 2013 |
|  | RV | AGGAGTATCTACAATATTTAC |
| *tet*(W) | FW | GAGAGCCTGCTATATGCCAGC | 168 | 64 | Jiang et al., 2013 |
|  | RV | GGGCGTATCCACAATGTTAAC |
| *tet*(K) | FW | TCGATAGGAACAGCAGTA | 169 | 61 | Jiang et al., 2013 |
|  | RV | CAGCAGATCCTACTCCTT |
| Aminoglycoside ARGs | | | | | |
| aph(2′′)-Ib | FW | CTTGGACGCTGAGATATATGAGCAC | 867 | 55 | Xu et al., 2015 |
|  | RV | GTTTGTAGCAATTCAGAAACACCCTT |
| *str*A | FW | CTTGGTGATAACGGCAATTC | 548 | 55 | Ouoba et al., 2008 |
|  | RV | CCAATCGCAGATAGAAGGC |
| *str*B | FW | ATCGTCAAGGGATTGAAACC | 509 | 56 | Ouoba et al., 2008 |
|  | RV | GGATCGTAGAACATATTGGC |
| *aad*A | FW | ATCCTTCGGCGCGATTTTG | 283 | 56 | Ouoba et al., 2008 |
|  | RV | GCAGCGCAATGACATTCTTG |
| *aad*E | FW | ATGGAATTATTCCCACCTGA | 386 | 50 | Ouoba et al., 2008 |
|  | RV | TCAAAACCCCTATTAAAGCC |
| *aac(*6ˊ)*-*Ib | FW | TATGAGTGGCTAAATCGAT | 395 | 55 | Huang et al., 2012 |
|  | RV | CCCGCTTTCTCGTAGCA |
| *arm*A | FW | CCGAAATGACAGTTCCTATC | 846 | 56 | Huang et al., 2012 |
|  | RV | GAAAATGAGTGCCTTGGAGG |
| *rmt*B | FW | ATGAACATCAACGATGCCCT | 769 | 56 | Huang et al., 2012 |
|  | RV | CCTTCTGATTGGCTTATCCA |
| Quinolone ARGs | | | | | |
| *qnr*S | FW | CCCCATGCCCGAAGTTATCA | 457 | 59 | Xu et al., 2015 |
|  | RV | ACTGCTTGGAGTGTGTTGGT |
| *aac(6**ˊ)-Ib-cr* | FW | ATATGCGGATCCAATGAGCAACGCAAAAACAAAGTTAG | 544 | 66 | Huang et al., 2012 |
|  | RV | ATAGCGAATTCTTAGGCATCACTGCGTGTTCGCTC |
| *qnr*A | FW | ATTTCTCACGCCAGGATTTG | 413 | 56 | Huang et al., 2012 |
|  | RV | GAGATTGGCATTGCTCCAGT |
| *gry*A | FW | CGATGTCGGTCATTGTTGGC | 455 | 61 | Xu et al., 2015 |
|  | RV | ATACCTACGGCGATACCGGA |
| *qnr*C | FW | TTCGATCGGACTGCTTGTGG | 438 | 59 | Xu et al., 2015 |
|  | RV | AACACATGGTGCAGGGGATT |
| *qnr*D | FW | GCTGGAGCTTGTCAGGGATT | 585 | 59 | Xu et al., 2015 |
|  | RV | TGCTGCGAGATATCATGCGT |
| *par*C | FW | GCCTAAACAACGCACGGAAA | 432 | 59 | Xu et al., 2015 |
|  | RV | TGACACGGGAGGTAACCAGA |
| *qnr*B | FW | TGGTGCTGTATGCACCGAAT | 453 | 58 | Xu et al., 2015 |
|  | RV | TCATCGCGCTGAAGAACTGT |
| Chloramphenicol ARGs | | | | | |
| *cat*I | FW | GGTGATATGGGATAGTGTT | 349 | 60 | Jiang et al., 2013 |
|  | RV | CCATCACATACTGCATGATG |
| *cat*II | FW | GATTGACCTGAATACCTGGAA | 567 | 60 | Jiang et al., 2013 |
|  | RV | CCATCACATACTGCATGATG |
| *cat*III | FW | CCATACTCATCCGATATTGA | 275 | 60 | Jiang et al., 2013 |
|  | RV | CCATCACATACTGCATGATG |
| *cat*IV | FW | CCGGTAAAGCGAAATTGTAT | 451 | 60 | Jiang et al., 2013 |
|  | RV | CCATCACATACTGCATGATG |
| *flo*R | FW | CGCCGTCATTCCTCACCTTC | 215 | 50 | Maynard et al., 2003 |
|  | RV | GATCACGGGCCACGCTGTGTC |
| Sulfonamide ARGs | | | | | |
| *sul*I | FW | CGCACCGGAAACATCGCTGCAC | 163 | 63 | Jiang et al., 2013 |
|  | RV | TGAAGTTCCGCCGCAAGGCTCG |
| *sul*II | FW | TCCGGTGGAGGCCGGTATCTGG | 191 | 63 | Jiang et al., 2013 |
|  | RV | CGGGAATGCCATCTGCCTTGAG |
| *sul*III | FW | TCCGTTCAGCGAATTGGTGCAG | 128 | 61 | Jiang et al., 2013 |
|  | RV | TTCGTTCACGCCTTACACCAGC |
| sulA | FW | TCTTGAGCAAGCACTCCAGCAG | 299 | 61 | Jiang et al., 2013 |
|  | RV | TCCAGCCTTAGCAACCACATGG |

**Supporting References**

Jiang L., Hu X. L., Xu T., Zhang H. C., Sheng D., Yin D. Q. (2013). Prevalence of antibiotic resistance genes and their relationship with antibiotic in the Huangpu River and the drinking water sources, Shanghai, China. *Science of the Total Environment*, 458-460, 267-272. doi: [10.1016/j.scitotenv.2013.04.038](https://doi.org/10.1016/j.scitotenv.2013.04.038)

Xu J., Xu Y., Wang H., Guo C., Qiu H., He Y., Zhang Y., Li X., Meng W. (2015). Occurrence of antibiotics and antibiotic resistance genes in a sewage treatment plant and its receiving river. *Chemosphere*,119, 1379-1385. doi: [10.1016/j.chemosphere.2014.02.040](https://doi.org/10.1016/j.chemosphere.2014.02.040)

Ouoba L., Lei V., Jensen L. B. (2008). Resistance of potential probiotic lactic acid bacteria and bifidobacteria of African and European origin to antimicrobials: Determination and transferability of the resistance genes to other bacteria. *International Journal of Food Microbiology*, 121(2), 217–224. doi: [10.1016/j.ijfoodmicro.2007.11.018](https://doi.org/10.1016/j.ijfoodmicro.2007.11.018)

Huang S. F., Dai W., Sun S., Zhang X. J., Zhang L. P. (2012). Prevalence of plasmid-mediated quinolone resistance and aminoglycoside resistance seterminats among carbapeneme non-susceptible Enterobacter cloacae. *Plos One*, 7(10), e47636. doi: [10.1371/journal.pone.0047636](https://doi.org/10.1371/journal.pone.0047636)

Maynard C., Fairbrother J. M., Bekal S., Sanschagrin F., Levesque R. C., Brousseau R., Masson L., Lariviere S., Harel J. (2003). Antimicrobial resistance genes in enterotoxigenic *Escherichia coli* O149:K91 isolates obtained over a 23-year period from pigs. *Antimicrobial Agents and Chemotherapy*, 47(10), 3214-3221. doi: [10.1128/AAC.47.10.3214-3221.2003](https://dx.doi.org/10.1128%2FAAC.47.10.3214-3221.2003)
