# Supplementary material for: A Comprehensive Research on Antibiotic Resistance Genes in Microbiota of Aquatic Animals
Source: Front Microbiol. 2018 Jul 26;9:1617. doi: 10.3389/fmicb.2018.01617 (PMC6070771; doi:10.3389/fmicb.2018.01617)
Supplement: Supplementary file 2 [file Table_2.docx]

**Table S2.** Primer sequences and amplification condition for qPCR

| Target genes | Sequence of primers (5–3’) | | Amplicon size (bp) | Annealing temperature (°C) for qPCR | Reference |
| --- | --- | --- | --- | --- | --- |
| *tet*S | FW | ATAGCGGTACAACGAAAACGG | 188 | 55 | Yi et al., 2010 |
|  | RW | GATTAGCAAAATAGCTCCATCCAA |  |  |  |
| *str*A | FW | ACCCTAAAACTCTTCAATGC | 152 | 60 | Faldynova et al., 2013 |
|  | RW | ACCCTAAAACTCTTCAATGC |  |  |  |
| *str*B | FW | GCTCGGTCGTGAGAACAATCT | 101 | 56 | Zhu et al., 2010 |
|  | RW | CAATTTCGGTCGCCTGGTAGT |  |  |  |
| *aad*A | FW | GCAGCGCAATGACATTCTTG | 282 | 55 | Madsen et al., 2000 |
|  | RW | ATCCTTCGGCGCGATTTTG |  |  |  |
| *flo*R | FW | ATTGTCTTCACGGTGTCCGTTA | 61 | 50 | Zhu et al., 2010 |
|  | RW | CCGCGATGTCGTCGAACT |  |  |  |
| *sul*I | FW | CGCACCGGAAACATCGCTGCAC | 163 | 62 | Pei et al., 2006 |
|  | RW | TGAAGTTCCGCCGCAAGGCTCG |  |  |  |
| *sul*II | FW | TCCGGTGGAGGCCGGTATCTGG | 191 | 62 | Pei et al., 2006 |
|  | RW | CGGGAATGCCATCTGCCTTGAG |  |  |  |
| 16S rRNA | FW | TGTGTAGCGGTGAAATGCG | 140 | 62 | Duan et al., 2009 |
|  | RW | CATCGTTTACGGCGTGGAC |  |  |  |

**Supporting references**

Duan, Q. J., Shang, S. Q., & Wu, Y. D. (2009). Rapid diagnosis of bacterial meningitis in children with fluorescence quantitative polymerase chain reaction amplification in the bacterial 16s rrna gene. *European Journal of Pediatrics,* *168*(2), 211-216. doi: [10.1007/s00431-008-0747-5](https://doi.org/10.1007/s00431-008-0747-5)

Faldynova, M., Videnska, P., Havlickova, H., Sisak, F., Juricova, H., & Babak, V., et al. (2013). Prevalence of antibiotic resistance genes in faecal samples from cattle, pigs and poultry. *Veterinární Medicína,58*(6), 298-304. [doi: 10.17221/6865-VETMED](http://dx.doi.org/10.17221/6865-VETMED)

Madsen, L., Aarestrup, F. M., & Olsen, J. E. (2000). Characterisation of streptomycin resistance determinants in danish isolates of salmonella, typhimurium. *Veterinary Microbiology,* *75*(1), 73-82.

Pei, R., Kim, S. C., Carlson, K. H., & Pruden, A. (2006). Effect of river landscape on the sediment concentrations of antibiotics and corresponding antibiotic resistance genes (arg). *Water Research,40*(12), 2427-2435. doi: [10.1016/j.watres.2006.04.017](https://doi.org/10.1016/j.watres.2006.04.017)

Yi, L., Mao, D. Q., Rysz, M., Zhou, Q. X., Zhang, H. J., & Lin, X., et al. (2010). Trends in antibiotic resistance genes occurrence in the haihe river, china. *Environmental Science & Technology,* *44*(19), 7220. doi: [10.1021/es100233w](https://doi.org/10.1021/es100233w)

Zhu, Y. G., Johnson, T. A., Su, J. Q., Qiao, M., Guo, G. X., & Stedtfeld, R. D., et al. (2013). Diverse and abundant antibiotic resistance genes in chinese swine farms. *Pnas,* *110*(9), 3435-3440. doi: [10.1073/pnas.1222743110](https://doi.org/10.1073/pnas.1222743110)

[7] Luo Y, Mao D, Rysz M, et al. Trends in antibiotic resistance genes occurrence in the Haihe River, China[J]. Environmental science & technology, 2010, 44(19): 7220-7225.

[8] Faldynova M, Videnska P, Havlickova H, et al. Prevalence of antibiotic resistance genes in faecal samples from cattle, pigs and poultry[J]. Vet Med Czech, 2013, 58: 298-304.

[9] Zhu Y G, Johnson T A, Su J Q, et al. Diverse and abundant antibiotic resistance genes in Chinese swine farms[J]. Proceedings of the National Academy of Sciences, 2013, 110(9): 3435-3440.

[10] Madsen L, Aarestrup F M, Olsen J E. Characterisation of streptomycin resistance determinants in Danish isolates of Salmonella Typhimurium[J]. Veterinary microbiology, 2000, 75(1): 73-82.

[11] Pei R, Kim S C, Carlson K H, et al. Effect of river landscape on the sediment concentrations of antibiotics and corresponding antibiotic resistance genes (ARG)[J]. Water research, 2006, 40(12): 2427-2435.

[12] Duan Q J, Shang S Q, Wu Y D. Rapid diagnosis of bacterial meningitis in children with fluorescence quantitative polymerase chain reaction amplification in the bacterial 16S rRNA gene[J]. European Journal of Pediatrics, 2009, 168(2):211-216.
